# Therapeutic Potential of Astrocyte Purinergic Signalling in Epilepsy and Multiple Sclerosis

**DOI:** 10.3389/fphar.2022.900337

**Published:** 2022-05-02

**Authors:** Paola Nobili, Weida Shen, Katarina Milicevic, Jelena Bogdanovic Pristov, Etienne Audinat, Ljiljana Nikolic

**Affiliations:** ^1^ Institute of Functional Genomics (IGF), University of Montpellier, CNRS, INSERM, Montpellier, France; ^2^ School of Medicine, Zhejiang University City College, Hangzhou, China; ^3^ Center for Laser Microscopy, Institute of Physiology and Biochemistry “Ivan Djaja”, University of Belgrade, Faculty of Biology, Belgrade, Serbia; ^4^ Department of Life Sciences, University of Belgrade, Institute for Multidisciplinary Research, Belgrade, Serbia; ^5^ Department of Neurophysiology, University of Belgrade, Institute for Biological Research Siniša Stanković, National Institute of Republic of Serbia, Belgrade, Serbia

**Keywords:** astroglia, disease, intercellular interaction, neuroinflammation, P2X7, P2Y1

## Abstract

Epilepsy and multiple sclerosis (MS), two of the most common neurological diseases, are characterized by the establishment of inflammatory environment in the central nervous system that drives disease progression and impacts on neurodegeneration. Current therapeutic approaches in the treatments of epilepsy and MS are targeting neuronal activity and immune cell response, respectively. However, the lack of fully efficient responses to the available treatments obviously shows the need to search for novel therapeutic candidates that will not exclusively target neurons or immune cells. Accumulating knowledge on epilepsy and MS in humans and analysis of relevant animal models, reveals that astrocytes are promising therapeutic candidates to target as they participate in the modulation of the neuroinflammatory response in both diseases from the initial stages and may play an important role in their development. Indeed, astrocytes respond to reactive immune cells and contribute to the neuronal hyperactivity in the inflamed brain. Mechanistically, these astrocytic cell to cell interactions are fundamentally mediated by the purinergic signalling and involve metabotropic P2Y1 receptors in case of astrocyte interactions with neurons, while ionotropic P2X7 receptors are mainly involved in astrocyte interactions with autoreactive immune cells. Herein, we review the potential of targeting astrocytic purinergic signalling mediated by P2Y1 and P2X7 receptors to develop novel approaches for treatments of epilepsy and MS at very early stages.

## Introduction

Neuroinflammation, an inflammatory response within the central nervous system (CNS), orchestrated by the cross-talk between CNS and immune system is now considered a key player in different neurological disorders, including epilepsy and multiple sclerosis (MS). It is characterized by the recruitment of immune cells to the CNS and also by a transformation of astrocytes and microglia into reactive profiles (Ransohoff, 2016; Yang and Zhou, 2019). These events have the primary role to maintain homeostasis by inducing the production and release of cytokines and chemokines that favour cell growth and survival. However, sustainment of inflammatory pathways leads to cellular dysfunctions actively contributing to the development and progression of chronic CNS disease (Vezzani et al., 2011; Xanthos and Sandkuhler, 2014; Yang and Zhou, 2019).

Epilepsy, with its incidence of ∼1%, represents one of the most common neurological disorders (Thijs et al., 2019). Evidence from both human patients and animal models demonstrated that neuroinflammation is not only a consequence but is actively involved in epileptogenesis and in seizure generation (ictogenesis) (Ravizza et al., 2008; Prabowo et al., 2013). For a long time, the study of epilepsy has been characterized by a neuro-centric view focused on the imbalance between excitatory and inhibitory transmissions that leads to neuronal hyperexcitability (Naylor, 2010; Bonansco and Fuenzalida, 2016). For this reason, current therapeutic strategies target mostly neuronal receptors and voltage-gated ion channels (Bialer et al., 2018). Epilepsy treatment however, remains still a challenge considering that ∼30% of patients do not respond to current antiseizure drugs (Janmohamed et al., 2020).

MS is a chronic inflammatory disease of the CNS, affecting 2.8 million people worldwide (Baecher-Allan et al., 2018). Understanding of MS pathology is characterized by the immuno-centric view focused on the immune cell infiltration into the CNS leading to the neuroinflammation and development of the multifocal inflammation, oligodendrocyte loss, demyelination, and axonal degeneration (Baecher-Allan et al., 2018; Dobson and Giovannoni, 2019). Current therapeutic strategies for MS treatment thus mostly focus on preventing and limiting immune cells trafficking into the CNS and modifying the immune cells responses (De Jager and Hafler, 2007; Rommer et al., 2019). However, immune cell-mediated inflammatory mechanisms alone, cannot explain neuronal degeneration without the CNS-resident cells component.

Great part in maintaining an optimal milieu for proper neuronal functioning rests with astrocytes. Indeed, astrocytes are positioned between blood vessels and synapses which allows them to fulfil metabolic and homeostatic maintenance functions (Parpura and Verkhratsky, 2012). This strategic location engages astrocytes in crosstalk with neurons, microglia, oligodendrocytes, other astrocytes, immune cells and blood vessels. Several studies have shown that neuroinflammation promoted astrogliosis changes the interaction between astrocytes and other cell types and alters the excitability of neuronal networks (Araque et al., 2014; Colombo and Farina, 2016; Vezzani et al., 2019; Sanz and Garcia-Gimeno, 2020; Audinat and Rassendren, 2021). Purinergic signalling, mediated by ATP and its breakdown products, is crucial in maintaining this astrocytic cell-to-cell communication (Franke et al., 2012) and increasing evidence have drawn considerable attention to an important role of astrocytic purinergic receptors (PRs) in epilepsy and MS from the initial stages of diseases (Narcisse et al., 2005; Dona et al., 2009; Franke and Illes, 2014; Rassendren and Audinat, 2016; Amadio et al., 2017; Brambilla, 2019; Domercq and Matute, 2019; Illes, 2020; Nikolic et al., 2020; Sidoryk-Wegrzynowicz and Struzynska, 2021). This opens possibility to target astrocytic purinergic signalling to develop non-neuronal and non-immune cell based therapeutic strategies for treatment of epilepsy and MS, respectively. These strategies would aim at better control of epilepsy and MS progression and even serve as a diseases-modifying entry points.

Considering the possibility to identify novel purine-based astrocyte-targeting treatments for epilepsy and MS, we here discuss the impact of astrocyte intercellular communication mediated by purinergic signalling on the pathogenic mechanisms underlying these two diseases. We focus on specific P2Rs, namely P2Y1R and P2X7R, which have been recently identified as key astrocyte receptors involved in epilepsy and MS, respectively.

### At the Interface of Astrocyte Purinergic Signalling and Neuroinflammation Properties of Astrocyte Purinergic Signalling

ATP is a predominant extracellular signalling molecule that mediates signalling among astrocytes, and between astrocytes and other cell types. In response to various physiological and pathological stimuli, astrocytes can release ATP through different ways, including exocytosis and large conductance channels, such as hemichannels, pannexin channels, volume-regulated anion channels and P2X7Rs (Coco et al., 2003; Franke et al., 2012; Bijelic et al., 2020). Furthermore, released ATP is rapidly converted into adenosine by a set of ectonucleotidases. Extracellular levels of adenosine are also controlled by astrocytes through the action of nucleoside membrane transporters and of the enzyme adenosine kinase (ADK) (Boison, 2012). Purine and its metabolites activate PRs, classified as adenosine P1Rs, and ATP/ADP P2Rs that are expressed in astrocytes and in a variety of cell types (Franke et al., 2012; Burnstock, 2020). P2Rs are divided into the cationic ion channel P2XRs and the G-protein-coupled P2YRs. Activation by purines (ATP, ADP, UDP) induces increase in intracellular Ca^2+^ in astrocytes: from extracellular sources for P2XRs and from intracellular sites for P2YRs (Burnstock, 2016).

### Astrocyte Purinergic Signalling Regulates Neuronal Activity

We and others have shown that selective photo-stimulation of hippocampal astrocytes expressing the light-sensitive Channelrhodopsin 2 (ChR2), increases Ca^2+^ signalling in astrocytes and subsequently triggers ATP release. This ATP excites inhibitory interneurons through the activation of P2Y1Rs but also activates P2Y1Rs expressed by astrocytes (Shen et al., 2017; Tan et al., 2017). This leads to the release of Ca^2+^ from intracellular stores and triggers glutamate release from astrocytes (Shen et al., 2017). In turn, this glutamate activates postsynaptic NMDA receptors on principal neurons and presynaptic metabotropic glutamate receptors (mGluR) in CA1 or presynaptic NMDA receptors in the dentate gyrus (Shen et al., 2017; Nikolic et al., 2018). Eventually, the degradation of ATP into adenosine leads to a delayed inhibition of principal neurons through the activation of adenosine A1Rs (Shen et al., 2017; Tan et al., 2017).

### ATP as an Inflammatory Signal and Astrogliosis

Besides its role in astrocyte intercellular communication, ATP may be an important signal contributing to astrogliosis, a common hallmark of neuropathological conditions that affects the physiological functions of astrocytes and leads to the appearance of new ones (Sofroniew, 2014; Escartin et al., 2019). Reactive astrocytes undergo morphological, molecular and functional changes (Sofroniew, 2020; Escartin et al., 2021) in diseases whereby context (pathogenic trigger, age, brain area, etc.) is important in determining reactive astrocyte phenotype and its consequences on the outcome of a disease (Anderson et al., 2016; Bonansco and Fuenzalida, 2016; Rothhammer et al., 2016; John Lin et al., 2017; Liddelow et al., 2017; Hartmann et al., 2019; Guttenplan et al., 2020; Borggrewe et al., 2021; Diaz-Castro et al., 2021; Escartin et al., 2021). Astrogliosis *in vivo* is observed after direct infusion of ATP or ADP (Hindley et al., 1994) or its structural analogue 2-methylthio ATP (2-MeSATP) in the brain (Franke et al., 1999), and *in vitro* after astrocyte challenge by ATP (Brambilla and Abbracchio, 2001) as measured by the hypertrophy of astrocytes and an increase in the glial fibrillary acidic protein (GFAP) immunoreactivity. These studies also showed that *in vivo* administration of P2Rs antagonists, suramine, reactive blue 2 and pyridoxal-phosphate-6-azophenyl-2, 4-disulphonic acid (PPADS) counteracted astrogliosis. Moreover, activation of P2Rs is linked to the transduction pathways that involve astrocyte production of various chemokines and cytokines, inflammatory mediators that all stimulate neuroinflammation (Franke et al., 2012). In context of epilepsy and MS, purine-mediated signalling could therefore be implicated in the appearance of reactive astrocytes and deregulation of their main homeostatic functions. Indeed, persistent astrogliosis and release of pro-inflammatory factors by reactive astrocytes can contribute to the BBB disruption that commonly occurs in epilepsy, and alterations in the two main astrocyte functions, such as potassium and glutamate buffering, leading to hyperexcitability (Abbott, 2000; Friedman et al., 2009; Marchi et al., 2014; Iori et al., 2016; Smith et al., 2018). Likewise, in MS purine-mediated signalling could be involved in appearance of reactive astrocytes with neurotoxic activities that release numerous inflammatory mediators that are responsible for the recruitment of peripheral inflammatory cells, activation of microglia and promotion of oligodendrocyte loss and demyelination (Schonrock et al., 2000; Stadelmann et al., 2002; Linker et al., 2010; Argaw et al., 2012; Prins et al., 2014; Rothhammer and Quintana, 2015; Brambilla, 2019; Wheeler and Quintana, 2019; Linnerbauer et al., 2020; Colombo et al., 2021).

### Astrocyte Purinergic Signalling Alterations and Their Impact on Epilepsy and MS

An enhancement in purinergic signalling is a common feature of different neuropathological conditions including epilepsy and MS (Franke et al., 2012; Rassendren and Audinat, 2016; Domercq and Matute, 2019). Considering that purinergic signalling is at the basis of astrocyte intercellular communication and associated with a reactive astrocyte profile, alterations in this purine-based signalling can impact on the neuroinflammatory response that occurs in both diseases. For this reason, strategies targeting astrocyte purinergic signalling are considered as promising new therapeutic treatments to better control the development and progression of both, epilepsy and MS.

### ATP and its Degradation Product Adenosine in Epilepsy

Increasing evidence indicates that astroglial-mediated purinergic signalling modulates seizure threshold and epileptogenesis (Cieslak et al., 2017). Adenosine has a well-known anticonvulsant action. However increased expression of the astroglial enzyme ADK, the major metabolic clearance route for adenosine, during epileptogenesis reduces the inhibitory tone of adenosine and consequently reduces the threshold for seizure generation (Boison and Steinhauser, 2018). Regarding ATP, Dossi et al. (Dossi et al., 2018) recently demonstrated an 80% increase of extracellular ATP during high potassium induced ictal discharges on slices obtained from resected tissues of TLE patients. This increase was pharmacologically blocked by inhibiting pannexin-1 channels and had anticonvulsive effects also in a mouse model of kainic acid (KA)-induced seizures.

### Ionotropic P2X7R in Epilepsy

Among ionotropic P2XRs, P2X7R is showed to be upregulated in epileptic mice and TLE patients, and P2X7R antagonism was able to reduce seizure severity and seizure-induced neuronal death (Jimenez-Pacheco et al., 2013), supporting the targeting of P2X7R as a potential therapeutic strategy for epilepsy. However, although P2X7R increased expression was well documented in neurons and microglia, its expression in hippocampal astrocytes remains highly controversial (Rassendren and Audinat, 2016). Even though, P2X7Rs may have a role in microglia-astrocyte communication in epilepsy. Activation of P2X7Rs on microglia induces their production and release of inflammatory signals (Campagno and Mitchell, 2021) that can promote astrogliosis. This can be a powerful mechanism of increasing a magnitude of inflammatory response in the CNS in epilepsy.

### Evidence Supporting Astrocytic P2Y1R as a Key Modulator of Epileptic Activity

Metabotropic P2Y1R is a promising astrocytic candidate to target in epilepsy as its downstream signalling is permanently active in the sclerotic hippocampus and its blockade restores normal neuronal activity in epilepsy (Alvarez-Ferradas et al., 2015; Nikolic et al., 2018; Wellmann et al., 2018). An increased P2Y1R expression was observed at the hippocampal level in both epilepsy animal models and TLE patients (Alves et al., 2017) and moreover, the activation of P2Y1R by means of ADP administration was sufficient to worsen seizure activity. Furthermore, it has been shown that P2Y1R is responsible for either proconvulsive or anticonvulsive effects depending if the receptor activation was occurring before or after SE induction (Alves et al., 2019a). There are some disagreements regarding the P2Y1R cell type specific expression. For example, P2Y1R expression is observed in microglia at a relatively weak level (Hidetoshi et al., 2012), and also in a cortical microglia in the epileptic brain (Alves et al., 2019b). Literature data, however, show that expression of P2Y1Rs is mainly detected in astrocytes (Franke et al., 1999; Jourdain et al., 2007; Kuboyama et al., 2011; Pascual et al., 2012; Delekate et al., 2014). Moreover, activation of P2Y1Rs mediates aberrant astrocytic Ca^2+^ activity and glutamate release that drives abnormal synaptic activity in epilepsy (Alvarez-Ferradas et al., 2015; Nikolic et al., 2018; Wellmann et al., 2018; Martorell et al., 2020). These data on expression and function support an argument on crucial role of astrocytic P2Y1Rs in mediating epileptic activity (Table 1).

**TABLE 1:** Examples of astrocyte P2Y1R- and P2X7R-mediated signalling in epilepsy and MS, respectively. Properties of astrocyte P2Rs-mediated signalling and effects of their blocking on pathophysiological events are showed for both diseases.

Astrocyte P2Y1R in Epilepsy				
Preparation/Model	P2Y1R signaling	P2Y1R block	Effect on neurons	Reference
epileptiform slices	↑P2Y1R	MRS 2179 MRS 2365	Prevention of abnormal synaptic frequency increase	Pascual et al. (2012)
	↑Glutamate release			
hippocampal slices/kindling epilepsy model	↑P2Y1R	MRS2179	Reversing of abnormal synaptic activity	Alvarez-Ferradas et al. (2015)
	↑Ca^2+^ signals			
	↑Glutamate release			
hippocampal slices/TLE model	↑P2Y1R	MRS 2179	Restoring of normal glutamatergic synaptic activity	Nikolic et al. (2018)
	↑Ca^2^ signals			
	↑Glutamate release			
hippocampal slices/kindling epilepsy model	↑P2Y1R	MRS2179	Rescuing of impaired synaptic plasticity	Martorell et al. (2020)
	↑Ca^2+^ signals			
	↑Glutamate release			
**Astrocyte P2X7R in MS**				
**Preparation/Model**	**P2X7R signalling**	**P2X7R block**	**Effect in MS**	**Reference**
brain sections/EAE	P2X7R null mice	P2X7R deficient	Reduction of GFAP expression	Sharp et al. (2008)
			Suppression of EAE development	
			Reduction of axonal damage	
brain sections/EAE	↑P2X7 expression	Brilliant blue G	Reduction of astrogliosis	Grygorowicz et al. (2016)
			Decrease of GFAP and S100β levels	
			Alleviating of disease symptoms	
brain sections/MS patients	↑P2X7 expression	Not studied	Astrogliosis near cortical lesions	Amadio et al. (2017)
Astrocyte culture/EAE (isolated CNS-infiltrated immune cells)	↑Ca^2^ signals	PPADS A438079	Blocking interaction of astrocytes and CNS-infiltrated immune cells	Bijelic et al. (2020)

### Astrocyte P2Y1R and Neuronal Activity in Epilepsy

**FIGURE 1:**
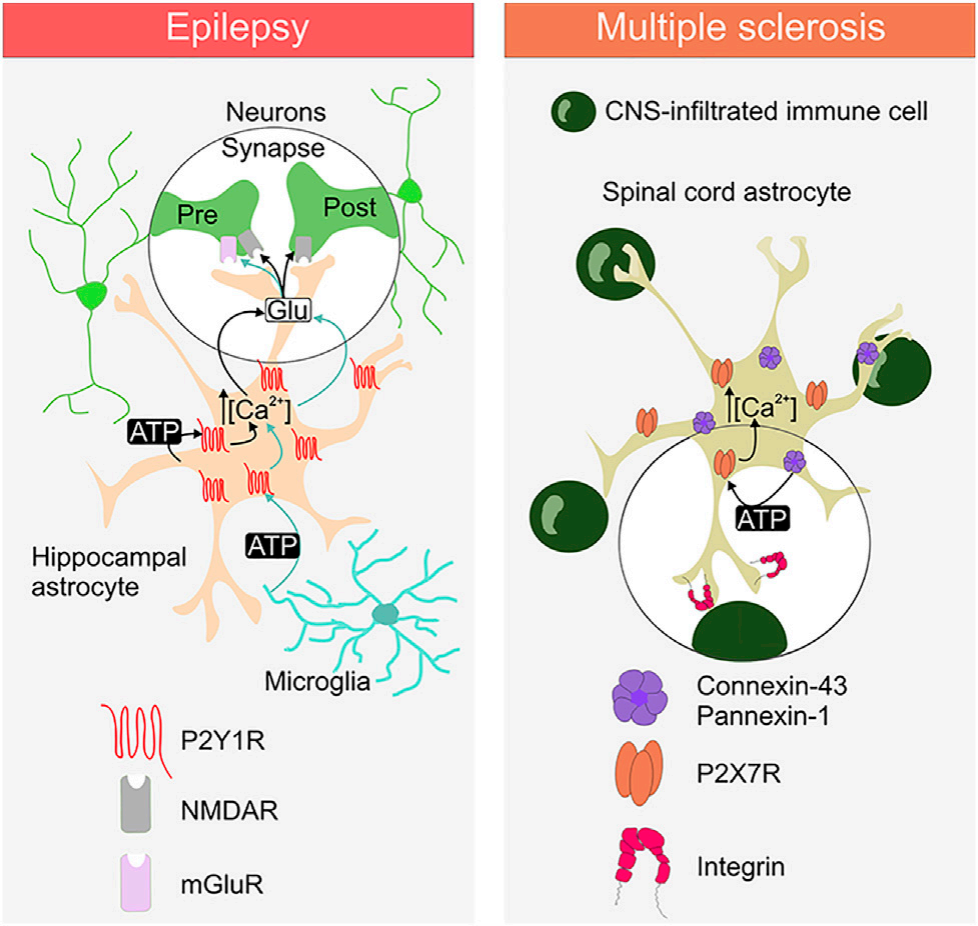
Purinergic signalling between astrocytes, neurons, microglia and CNS-infiltrated immune cell in epilepsy and multiple sclerosis. The ATP release and the involvement of the specific astrocytic purinergic receptor (PR) type is depicted for the diseases. Activation of astrocytic P2Y1R and P2X7R induce the rise in intracellular Ca^2+^. Activation of P2Y1R promotes the release of glutamate, which acts on the presynaptic and postsynaptic glutamate receptors (NMDAR and mGluR). Activation of P2X7Rs is mediated by the release of ATP through connexin-43 hemichannels and/or pannexin-1 channels. Integrins are depicted as a putative link to the astrocytic P2X7R activation *via* connexin-43/pannexin-1-derived ATP.

We recently showed that the pro-inflammatory cytokine tumor necrosis factor-α (TNFα), which is increased during seizure activity, is able to trigger a Ca^2+^-dependent glutamate release from astrocytes that boosts excitatory synaptic activity in the hippocampus of a TLE mouse model (Figure 1). We demonstrated that this mechanism involves an autocrine activation of P2Y1Rs by astrocyte-derived ATP/ADP. Indeed, reducing extracellular ATP/ADP with apyrase or blocking P2Y1Rs normalize glutamate release from astrocytes and restore normal synaptic activity during epileptogenesis (Nikolic et al., 2018). Furthermore, ATP can evoke a Ca^2+^-dependent glutamate release from astrocytes and trigger slow inward current (SIC) in neurons (Perea and Araque, 2005) leading to the synchronization of neuronal activity in the hippocampus (Angulo et al., 2004; Fellin et al., 2004). Activation of astrocytic P2X7Rs and P2Y1Rs do not mediate SIC appearance in physiological conditions (Fellin et al., 2006; Shigetomi et al., 2008). However, in epilepsy P2Y1-dependent glutamate release from astrocytes increases SIC frequency in CA1 neurons that can promote neuronal synchronisation (Alvarez-Ferradas et al., 2015), (Figure 1). Likewise, endogenously released ATP acting through astrocytic P2Y1Rs contributes to Ca^2+^ wave propagation of these glial cells (Bowser and Khakh, 2007) and it can play a role in promoting synchronized neuronal activity in epilepsy. These data support the importance of astroglial P2Y1R-mediated signalling in the epileptogenic process and suggest that purinergic therapeutic strategies could be useful in suppressing or ameliorating epileptic activity.

### P2Y1R-Mediated Astrocyte-Microglia Crosstalk in Epilepsy

TNFα can trigger the astrocyte ATP-P2Y1R loop (see above) in the dentate gyrus in the context of epilepsy (Nikolic et al., 2018) and maybe also in the context of experimental autoimmune encephalitis (EAE) (Santello et al., 2011;Habbas et al., 2015). Whereas the source of TNFα in these inflammatory conditions is probably microglia (Zhang et al., 2014), microglial cells can also activate astrocyte P2Y1R-mediated signalling directly by releasing ATP. Alain Bessis and colleagues found that LPS application triggers, within few minutes in acute hippocampal slices, ATP release from microglia that activates astrocytic P2Y1Rs. This leads to the glutamate release from astrocytes that increases excitatory postsynaptic current (EPSC) frequency in CA1 pyramidal neurons through presynaptic mGluR activation. Moreover, activation of this microglia-astrocyte purinergic signalling promotes the generation of epileptiform activities (Figure 1) (Pascual et al., 2012). These observations reinforce the idea that purinergic signaling is critical in astrocyte-microglia crosstalk under various pathological conditions (Franke et al., 2012; Agostinho et al., 2020; Illes et al., 2020; Matejuk and Ransohoff, 2020; Pietrowski et al., 2021).

### Astrocyte Purinergic Signalling in MS

Although less examined compared to epilepsy, astrocyte purinergic signalling can also be a critical contributor to the pathology of MS from its early stages and can also affect the course of the disease. Indeed, screening of selected P1R, P2XR and P2YR genes revealed that changes in their expression occur along with the different phases of the EAE development (Jakovljevic et al., 2017). The same study showed a decrease in the expression of ectonucleoside triphosphate diphosphohydrolase 2 (NTPDase2) in EAE, an ATP hydrolysing enzyme preferentially expressed in astrocytes. Furthermore, purine-based signalling mediates communication between astrocytes and oligodendrocytes (Fields and Burnstock, 2006; Ishibashi et al., 2006; Welsh and Kucenas, 2018; Agostinho et al., 2020) which may be critical in neuroinflammatory processes in MS. ATP release during neuronal activity induces astrocytes to release the promyelinating cytokine leukemia inhibitory factor (LIF), which promotes oligodendrocyte development (Ishibashi et al., 2006). However, others demonstrated that mechanical stimulation of astrocytes increases ATP release and ATP evokes Ca^2+^ signals in oligodendrocyte progenitor cells (OPCs) by a mechanism involving the activation of P2Y1Rs and P2X7Rs (Agresti et al., 2005; Hamilton et al., 2010). Moreover, over-activation of P2X7Rs in oligodendrocytes causes cell death due to the high cytosol Ca^2+^ levels, which contributes to demyelinating diseases. Considering that neurotoxic astrocytes contribute to the oligodendrocyte death in MS (Liddelow et al., 2017), targeting purine-based astrocyte-oligodendrocyte communication, could be a promising approach to prevent demyelination in this disease.

### Evidence Supporting P2X7R as a Key Astrocytic Target in MS

Ionotropic P2X7R could be a promising astroglial candidate to target in MS as this receptor is upregulated in astrocytes, its blockade reduces astrogliosis (Narcisse et al., 2005; Grygorowicz et al., 2016; Amadio et al., 2017), and it is involved in the communication between astrocytes and components of the immune system in this disease (Table 1). Although characterized as dominantly expressed by microglia, P2X7R is also expressed and functional in astrocytes in specific areas (Oliveira et al., 2011; Illes, 2020). Within CNS lesions of patients with MS, P2X7R expression is upregulated in reactive astrocytes surrounding infiltrated immune cells. The same study demonstrated that a treatment with the proinflammatory cytokine interleukin 1β increases mRNA expression of P2X7R, and also increases P2X7R-dependent Ca^2+^ response of human fetal astrocytes (Narcisse et al., 2005).

### P2X7R and Astrogliosis in MS

Previous research showed alterations in P2X7R expression in reactive astrocytes in the inflamed CNS. For example, P2X7Rs are up-regulated in reactive astrocytes in the frontal cortex of patients with MS (Amadio et al., 2017), while in EAE reactive astrocytes show increase in P2X7Rs immunoreactivity (Grygorowicz et al., 2016). In fact, blockade of P2X7Rs by Brilliant blue G administration in rats with EAE, decreases astrogliosis and alleviates disease symptoms (Grygorowicz et al., 2016). Furthermore, P2X7R deficiency supresses EAE development in P2X7R KO mice, reduces astroglial activation and axonal damage, but does not influence immune cell infiltration into the CNS (Sharp et al., 2008). Interestingly, P2X7Rs co-immunoprecipitate with pannexin-1 channel in astrocytes (Silverman et al., 2009), and pannexin-1 KO mice display a delay in the onset of the EAE clinical signs (Lutz et al., 2013). In this modality, pannexins-1 release ATP that activates P2X7Rs and further recruit components of the inflammasome.

### Astrocyte P2X7R and Interaction With CNS-Infiltrated Immune Cells

In addition to signalling through the release of ATP to communicate with other types of neural cells (Franke et al., 2012), astrocytes utilize this purine-based signalling also to interact with CNS-infiltrated immune cells (Bijelic et al., 2020). Our recent study showed that astrocyte interaction with a nearby CNS-infiltrated immune cells requires astrocytic P2X7R- and not P2Y1R-dependent signalling (Bijelic et al., 2020). Furthermore, the presence of CD4^+^ autoreactive immune T cells promotes astroglial hemichannel and or/pannexin channel-controlled ATP release that activates P2X7Rs and causes increase in the cytosolic Ca^2+^ in these glial cells (Bijelic et al., 2020). Initial step promoting P2X7Rs activation in astrocytes upon encountering autoreactive CD4^+^ T cells, can involve integrins (Figure 1), as integrin engagement has been linked to the astrocyte P2X7R activation *via* connexin-43 hemichannel and pannexin-1 channel release of ATP (Henriquez et al., 2011; Alvarez et al., 2016). Integrins are adhesion molecules that mediate cell-to-cell interaction, they are expressed on neural and immune cells and implicated in pathology of MS (Archelos et al., 1999). Moreover, integrin-inhibiting therapies such as natalizumab are currently in use for treatment of MS to reduce relapses in patients (Slack et al., 2022). This consideration linking astrocytic P2X7Rs with integrins pertains to the potential role of this PR as an astroglia-targeting therapeutic candidate for treatment of MS, to control interaction between astrocytes and CNS-inflitrated immune cells to reduce neuroiflammation in this disease (Figure 1).

## Conclusion

Deciphering astrocytic purinergic signalling and neuroinflammation in epilepsy and MS is a challenging task considering the cell types involved and the communication pathways operating in the inflamed CNS in these diseases. ATP release and astroglial-mediated purinergic signalling are crucial mechanisms through which astrocytes communicate between themselves, with neurons in epilepsy and with autoreactive immune cells in EAE (Figure 1), making them suitable targets for designing innovative disease-modifying strategies. In case of epilepsy, this would mean developing effective therapies that will not just target neurons, but also astrocytes, which by themselves provide conditions for proper neuronal functioning. Similarly in MS, demyelination and neuronal loss, could be rescued by effective controlling and targeting astrocyte interaction with autoreactive immune cells. Quite possibly, saving astrocyte protective functions would be a far more effective strategy to save neurons from damage in both, epilepsy and MS as virtually every aspect of brain function involves a neuron-astroglial partnership. We pointed toward P2Y1R and P2X7R as two potential astrocytic therapeutic candidates for treatment of epilepsy and MS, respectively. Common for both astrocytic receptor types is their involvement in autocrine loops, which further confirms that astrocyte targeting can be exploited to design effective therapeutic strategy. Further understanding of the purine-based signalling in mediating astrocyte cell to cell interactions holds a promise to develop new strategies for treatment of these neurological disorders.
